# Autoimmunity-related demyelination in infection by Japanese encephalitis virus

**DOI:** 10.1186/1423-0127-18-20

**Published:** 2011-02-28

**Authors:** Yu-Fen Tseng, Chien-Chih Wang, Shuen-Kuei Liao, Ching-Kai Chuang, Wei-June Chen

**Affiliations:** 1 Graduate Institute of Biomedical Sciences, Chang Gung University, Kwei-San, Tao-Yuan 33332, Taiwan; 2 Graduate Institute of Clinical Medical Sciences, Chang Gung University, Kwei-San, Tao-Yuan 33332, Taiwan; 3 Cancer Immunotherapy Center, Taipei Medical University, Taipei 11031, Taiwan; 4 Department of Public Health and Parasitology, Chang Gung University, Kwei-San, Tao-Yuan 33332, Taiwan

## Abstract

Japanese encephalitis (JE) virus is the most common cause of epidemic viral encephalitis in the world. The virus mainly infects neuronal cells and causes an inflammatory response after invasion of the parenchyma of the brain. The death of neurons is frequently observed, in which demyelinated axons are commonly seen. The mechanism that accounts for the occurrence of demyelination is ambiguous thus far. With a mouse model, the present study showed that myelin-specific antibodies appeared in sera, particularly in those mice with evident symptoms. Meanwhile, specific T cells proliferating in response to stimulation by myelin basic protein (MBP) was also shown in these mice. Taken together, our results suggest that autoimmunity may play an important role in the destruction of components, *e.g*., MBP, of axon-surrounding myelin, resulting in demyelination in the mouse brain after infection with the JE virus.

## Background

Japanese encephalitis (JE) is a significant mosquito-borne viral disease that causes a great number of encephalitic epidemics particularly in Asian countries [1]. The JE virus is a member of the *Flavivirus*, belonging to the family Flaviviridae; the genome is composed of single-stranded, positive-sense RNA of approximately ~11,000 nucleotides in length, and contains a single open reading frame (ORF) that encodes 10 proteins including 3 structural and 7 non-structural ones [2]. In general, JE viral infection is estimated to cause about a 25%~30% case-fatality rate [3]. More importantly, permanent neuropsychiatric sequelae related to JE are reported to appear in up to 50% of survivors [4].

The JE virus, through mosquito bites, is hypothetically amplified in dermal tissues and then lymph nodes via migration of dendritic (Langerhans) cells prior to invasion of the central nervous system (CNS) [5]. In most cases, JE patients clinically appear as having encephalomyelitis involving the cortex, subcortex, brainstem, and spinal cord [4,6], mostly presenting with such clinical symptoms as headaches, vomiting, an altered mental state, as well as dystonia, rigidity, and a characteristic mask-like facies [7]. Surviving patients may slowly regain neurological function over several weeks despite only one-third of cases recovering normal neurological functions [8]. Meanwhile, a proportion of them may exhibit clinical sequelae including motor weakness, intellectual impairment, and seizure disorders [3,4]. Specifically, intellectual involvement is noted in 30% of cases, speech disturbance in 34%, and motor deficits in 49% of such patients [8]. It was reported that the JE virus enters the CNS by way of an impaired blood-brain barrier (BBB) [9], presumably carried by infected peripheral blood mononuclear lymphocytes (PBMCs) [10,11].

In the CNS of JE patients, the virus may infect a variety of brain tissues with a characteristic pattern of mixed intensity or hypodense lesions including the thalamus, basal ganglia, and midbrain [6]. Clinically, movement disorders are frequently shown in patients who survive the acute phase of JE [12], implying that sensorimotor neuropathy eventually occurs. It is now known that encephalitis associated with flaviviral infections may cause Guillain-Barré-like syndrome, showing a demyelinating feature in sensorimotor tissues of the brain [13]. This suggests that demyelination is an important step causing disruption of motor coordination during viral infection [14].

Either necrosis or apoptosis causes death of neurons infected by encephalitic arthropod-borne viruses [15,16]. In addition, acute neuronal apoptosis was connected to inflammatory and demyelinating disease of the CNS in a rat model of multiple sclerosis [17]. In fact, we previously observed that demyelination commonly occurs in the mouse brain infected by the JE virus. Nevertheless, how demyelination occurs in brains infected with this virus remains ambiguous. In this study, we provide experimental evidence showing the role of immune responses in the occurrence of demyelination. This provided insights for further understanding of the pathogenesis of JE virus infection, especially in terms of movement disorders.

## Methods

### Virus and animals

The T1P1 strain of the JE virus used in this study is a local strain from Taiwan; it was isolated from the mosquito, *Armigeres subalbatus *[18]. The virus was propagated in C6/36 cells, and titrated with BHK-21 cells by means of plaque assays following the description in one of our previous reports [19]. In total, 21 female ICR mice at 4~6 weeks old were used in this study. Mice in the study group were intravenously injected with a dose of 1 × 10^6 ^plaque-forming units (PFU)/mouse of a viral suspension diluted with phosphate-buffered saline (PBS, pH 7.4) to a final volume of 100 μl. Those mice used as the control were inoculated with a virus-free solution diluted with cell culture medium. The movements and body coordination of inoculated mice were monitored daily for 3 wk. Mice with or without evident symptoms (movement disorders, mostly rigidity of the hindlimbs) were sacrificed to harvest serum samples for serological investigations and brain tissues for light and electron microscopy.

### Frozen sectioning

Brain tissues were dissected out from mice inoculated with and without the virus suspension. A part of the brain was prepared for frozen sectioning to investigate the histopathology and immunohistochemistry; the other part was used for a virological examination. For frozen sectioning, brain tissues embedded in tissue-freezing medium (Jung, Nussloch, Germany) were transiently frozen in liquid nitrogen and cut with a cryomicrotome (CM3050S; Leica, Mannheim, Germany). Sections 7~8 μm thick were collected and placed on slides coated with Silane S (Muto Pure Chemicals, Tokyo, Japan), then fixed in cold acetone for 15 min before being stained.

### Hematoxylin and eosin (H&E) staining

Frozen sections placed on slides were fixed in 24% formalin for 30 s and then washed with distilled water. Sections were subsequently stained with hematoxylin for 1 min. After being washed with distilled water, sections were dipped in 0.25% ammonia for 10 s and subsequently stained with eosin for 20 s after another wash with distilled water. Sections were then dehydrated with 95% and absolute ethanol in sequence. Sections were subsequently infiltrated with xylene and mounted in Entellan^® ^(EMS, Hatfield, PA, USA).

### Luxol fast blue staining

This staining protocol is used to stain myelin/myelinated axons. The approach for staining in this study followed a previously described method [20]. Briefly, frozen sections were immersed in 95% alcohol for 5 min before being stained with a 0.1% Luxol blue solution and a 0.1% Cresyl echt violet solution. The results show deep blue for myelin, violet for nuclei, and pale green for erythrocytes.

### Electron microscopy

Brain tissues dissected from mice were immediately fixed with 2% (v/v) glutaraldehyde in 0.1 M cacodylate buffer overnight at 4 °C. Tissues were subsequently postfixed in 1% (w/v) osmium tetroxide in 0.1 M cacodylate buffer for 2 h at room temperature and then washed with 0.2 M cacodylate buffer 3 times. After washing, tissues were dehydrated through an ascending graded series of ethanol and ultimately were embedded in Spurr's resin (EMS) and polymerized at 70°C for 72 h. Trimmed tissue blocks were sectioned with an ultramicrotome (Reichert Ultracut R, Leica, Vienna, Austria). Thin sections were sequentially stained with saturated uranyl acetate in 50% ethanol and 0.08% lead citrate. Selected images were observed and photographed under an electron microscope (JEOL JEM-1230, Tokyo, Japan) at 100 kV.

### Reverse-transcriptase polymerase chain reaction (RT-PCR)

Brain tissues were homogenized with minimum essential medium (MEM), from which RNA was extracted using the Trizol^® ^reagent (Invitrogen, Carlsbad, CA). Primers and reaction conditions used for the subsequent RT-PCR are described in our previous report.^11 ^The PCR product was a ~291-bp fragment of the envelope (E) protein of the JE virus, that could be seen by electrophoresis on a 2% (w/v) agarose gel containing 10 μl ethidium bromide (1 mg/ml in RNase-free water).

### Enzyme-linked immunosorbent assay (ELISA)

An indirect ELISA was used to detect specific MBP immunoglobulin G (IgG) antibodies in this study. Initially, 5 μl of mouse MBP (Sigma, St. Louis, MO, USA) was coated on 96-well ELISA plates, followed by blocking with 1% bovine serum albumin (BSA). Mouse serum diluted to 1:50 in 1% BSA was added to each well of the plates. After the plates were washed, a sheep anti-mouse IgG antibody conjugated with horseradish peroxidase (HRP) (GE Healthcare, Piscataway, NJ, USA) diluted to 1: 2000 in 1% BSA was added to the wells. After another wash, the ABTS peroxidase substrate (KPL, Gaithersburg, MD, USA) was added to the wells and incubated for 10~15 min. Optical density (OD) values of each well of the plates were read at a wavelength of 405 nm.

### Isolation of splenocytes

To isolate splenocytes, dissected spleens were placed in a 6-cm dish filled with RPMI culture medium (GIBCO^®^, Grand Island, NY, USA) containing 10% fetal calf serum (FCS), 1% antibiotic-antimycotic (GIBCO^®^), and 50 mM 2-mercaptoethanol (2-ME) (Sigma). The spleen was subsequently disaggregated with a 23G needle to separate splenocytes which were moved into a 50-ml centrifuge tube and then centrifuged at 3000 rpm and 4°C for 10 min. After discarding the supernatant, erythrocytes in the pellet were removed by hypotonic lysis with 1 ml H_2_O. Splenocytes in the pellet were resuspended in 7 ml PBS. After centrifugation, the supernatant was discarded. Then, 10 ml of Earle's balanced salt solution (EBSS) (Biological Industries, Beit Haemek, Israel) was added to the tube for further centrifugation. Subsequently, RPMI culture medium was added to the tube to replace the supernatant. Ultimately, numbers of isolated splenocytes were counted with a hematocytometer.

### T cell proliferation

Splenocytes isolated from mice were cultured (2 × 10^5 ^cells/well) with RPMI medium. In total, 50 μg/ml mouse MBP was added to each well of the plates, incubated at 37°C with a 5% CO_2 _atmosphere for 72 h, and then pulsed with 1 μCi [^3^H]-thymidine (Perkin Elmer, Waltham, MA, USA) for 18 h before being harvested. Radioactivity was determined directly in the plate with a β-counter (TopCount, NXT™, Packard Instrument Co., Meriden, CT, USA). Proliferation was expressed as a stimulation index (SI) that was estimated by a ratio of counts in each well cultured with the MBP antigen over that cultured with MBP-free medium.

### Statistical analysis

Comparisons of the two means were analyzed by Student's *t*-test at a significance level of 5%.

## Results

### Detection of JE virus in the mouse brain

**Figure 1:**
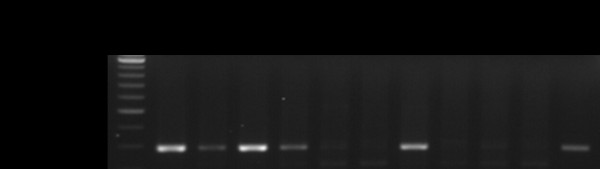
**Detection of viral RNA by RT-PCR in brains of mice inoculated with Japanese encephalitis virus**. The size of the amplified fragment was estimated to be 291 bp. Viral RNA was observed in all mice with evident clinical symptoms (m1, m2, and m4). A mouse (c1) inoculated with culture medium was used as the control. In addition, viral RNA was detected from both the cerebrum (marked with B) and the cerebellum (marked with b) of brains of infected mice. The lane marked "v" is the positive control taken from a cultured virus suspension.

Five mice were chosen to detect viral RNA extracted from either the cerebrum or cerebellum. Two (m1 and m2) with evident symptoms and one with slight symptoms (m4) were detected to be positive except for the cerebellum of m4. Both parts of the brain in a control and one inoculated mouse with extremely slight symptoms were found to be negative. Those positive for viral RNA always showed higher amounts of viral RNA in the cerebrum that in the cerebellum (Figure 1).

### Pathologic features of the mouse brain with JE viral infection

**Figure 2:**
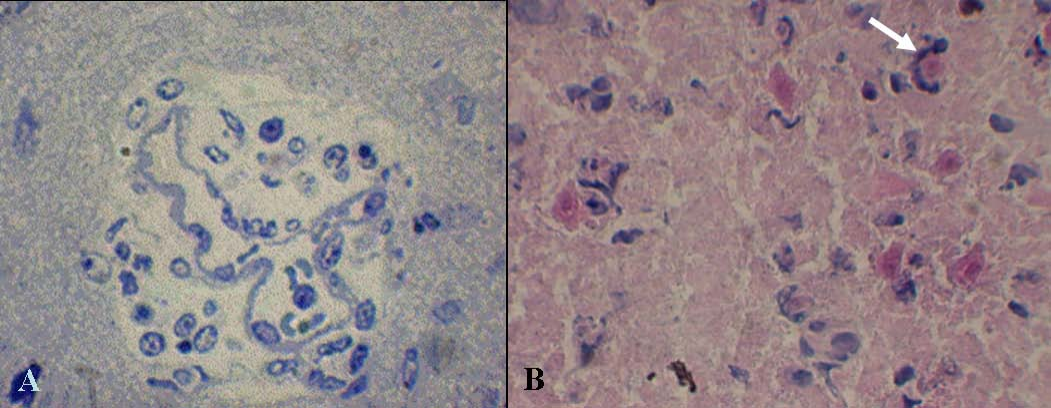
**Pathological changes with severe inflammation in a mouse brain infected with Japanese encephalitis virus**. **(A) **Inflammatory infiltrate around the vessel in the brain. The blood vessel is congested with inflammatory cells. **(B) **Degenerating neurons (pink) shown in the brain of symptomatic mice are being engulfed by phagocytes (arrow). Hematoxylin and Eosin staining. Original magnification: × 400.

**Figure 3:**
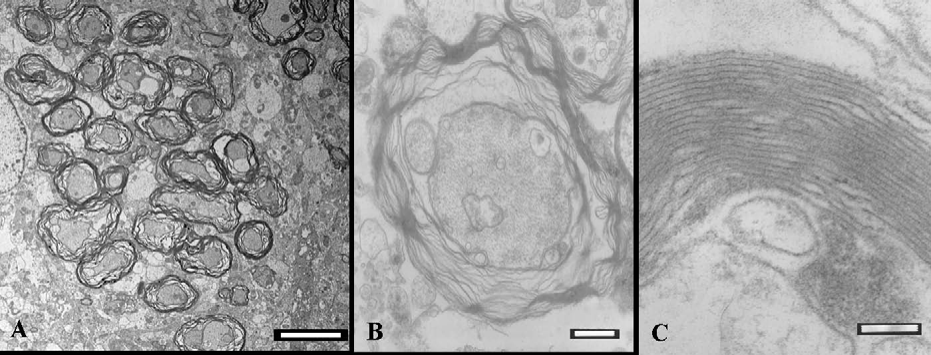
**Demyelination shown in the brain of mice infected with the Japanese encephalitis virus**. **(A) **Severely demyelinating axons extensively distributed in the brain, primarily the cerebrum, of symptomatic mice. **(B) **Demyelinated axons present a loose composition of myelin. **(C) **Normal axons were surrounded by a myelin sheath that was condensed with intraperiodic lines. Scale bar = 5 μm for A, 500 nm for B, and 100 nm for C.

Hindlimbs of inoculated mice with symptoms frequently appeared paralytic, an important sign of infection by JE virus in mice. In general, severe inflammation usually occurred as shown in the brain of JE virus-inoculated mice. Histological evidence showed that vessels were frequently congested by increased numbers of inflammatory cells in and around capillaries of the brain, particularly the cerebrum (Figure 2A). Degenerating neurons were commonly seen in the brain of symptomatic mice; they were usually engulfed and were removed by phagocytes (Figure 2B). Numerous demyelinating axons were primarily distributed in the cerebrum of symptomatic mice (Figure 3A), where demyelination presented a loose composition of myelin (Figure 3B). In contrast, normal axons were surrounded by the myelin sheath that was condensed with intraperiodic lines (Figure 3C).

### MBP-specific antibody in sera of JE virus-infected mice

**Figure 4:**
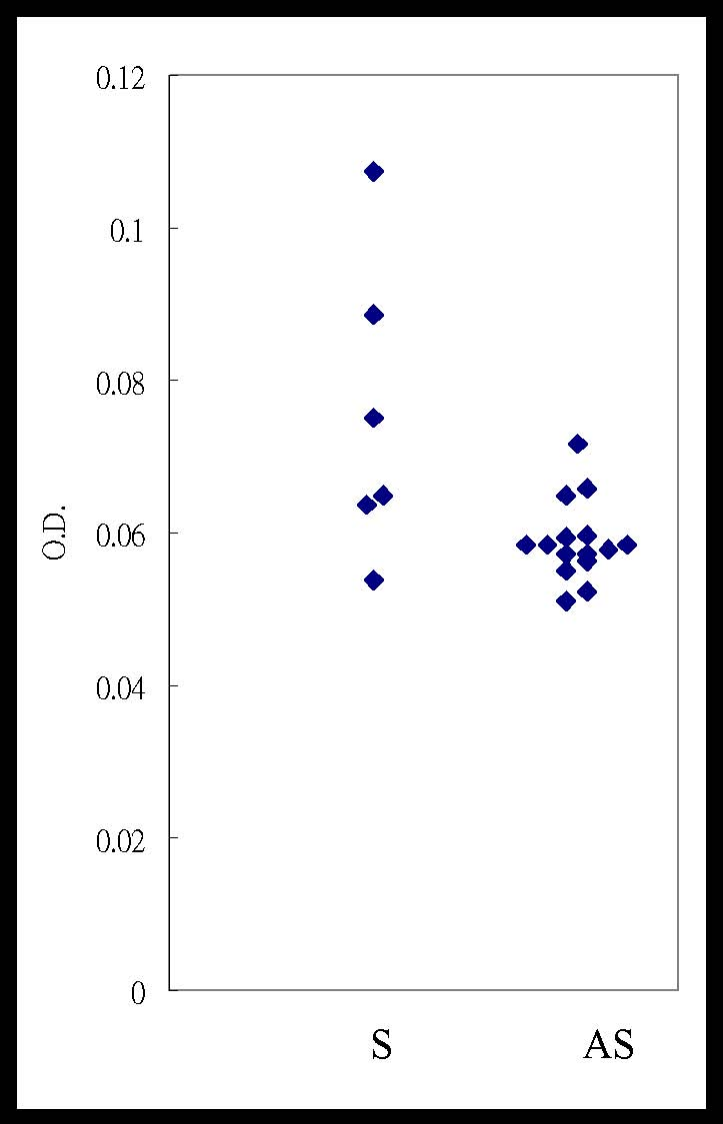
**Detection of an anti-MBP antibody in mouse sera collected from JE virus-infected mice**. Average titers of the anti-MBP antibody (IgG) from mice with impaired movement were significantly higher than those in asymptomatic ones (Student's *t*-test, *p *< 0.05). S, Sera from symptomatic mice; AS, sera from asymptomatic mice.

Among 21 mice chosen in this experiment for inoculation with a JE viral suspension, 15 were asymptomatic while the other 6 showed symptoms with movement disability after a period of 21 d. Of these, 3 (3/6; 50%) of the symptomatic and 1 (1/15; 6.67%) of the asymptomatic mice were positive for the MBP-specific antibody (Table 1). The OD value from mice with symptoms was 0.076 ± 0.019, which was significantly higher than that from the asymptomatic mice (0.057 ± 0.005) (Student's *t *test; *p *< 0.05) (Figure 4).

**Table 1:** Specific IgG antibody to myelin basic protein (MBP) detected in Japanese encephalitis virus-infected mice that did or did not exhibit symptoms during a period of 21 days of observation

Symptoms	Number of observations	**Number positive**^**†**^	Mean ± SD	Statistics*
+	6	3 (50%)	0.076 ± 0.019	*p *< 0.05
-	15	1 (6.67%)	0.057 ± 0.005	

^† ^Being positive was determined by the criterion of being higher than the sum of the average optical density (OD) value and 2 standard deviations (SDs) of the control group.

* Student's *t*-test at a 5% level of significance.

### MBP-specific T cell proliferation in response to JE viral infection

**Figure 5:**
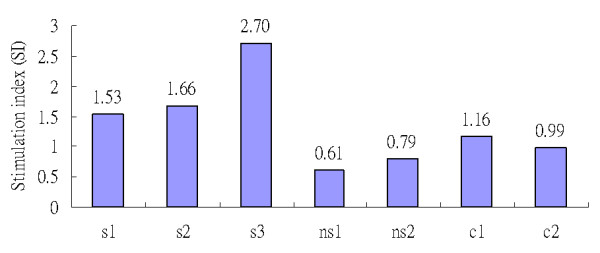
**T-cell proliferation in mice infected with the Japanese encephalitis virus after treatment with 50 μg/ml of the myelin basic protein (MBP)**. A stimulation index (SI) was used to express the efficacy of T-cell proliferation for each mouse compared to that from a mouse inoculated with culture medium only. Mouse s1~s3: mice with symptoms of movement disability; Mouse ns1~ns2: mice without evident symptoms.

To assess proliferation of specific T cells, mice infected with the JE virus were administrated 50 μg/ml MBP. The results showed that stimulation indexes (SIs), used to express the efficacy of T-cell proliferation, for three mice with symptoms (s1~s3) were 1.53, 1.66, and 2.70, respectively. In contrast, values were 0.61 and 0.79, respectively, for the two asymptomatic mice and 1.16 and 0.99, respectively, for the two control mice (inoculated with culture medium) (Figure 5). It was shown that higher SIs generally occurred in symptomatic mice compared to control and asymptomatic mice.

## Discussion

In a mouse model, intravenously inoculated JE virus migrates into the brain within 2 d after inoculation, causing the brain to become infected by the invading virus [10]. Both the cerebrum and cerebellum are frequently infected; in most cases, the cerebrum becomes infected earlier and more intensely [9]. Due to impairment of the BBB, alterations of tight junctions of capillaries in the CNS are believed to be the entrance route of inflammatory cells into the parenchyma of the brain [11,21]. This usually results in inflammation of the CNS [8] and causes cellular destruction as well [22].

The present results showed that demyelination commonly occurs in the brain of mice with JE virus infection. Demyelination is a common feature in the brain that is infected by encephalitis viruses as seen in patients with HIV infection [23]. It is the process by which axons lose myelin that normally serves as an insulator, resulting in loss of balance and coordination, although it may vary among patients [24]. While the causes of demyelination in the CNS remain unclear, various aspects were widely investigated such as in multiple sclerosis (MS) and viral infections, *e.g*., canine distemper virus [25] and mouse hepatitis virus [26]. Despite further evidence being expected, MS usually results in major disability and is now linked to viral infection, most likely Epstein-Barr virus (EBV) [27].

In the CNS of mammals, oligodendrocytes, constituting glial cells with microglia and astrocytes, are myelin-forming cells [28]. Injury to oligodendrocytes which end up undergoing apoptosis was postulated to be responsible for myelin destruction and subsequent demyelination [29]. Our observations showed that the JE virus normally infects neurons and astrocytes. However, most oligodendrocytes in the brains of those mice remained intact and uninfected [9]. Presumably, factors other than virus-induced oligodendrocyte damage may play an essential role in the occurrence of demyelination. Neuronal death which is widely seen in JE virus-infected mouse brain is probably important for induction of axonal injury and demyelination [30]. Many other mosquito-borne encephalitic viruses such as Sindbis virus (SV) are reported to be associated with death of neuronal cells [15], which possibly is the first step in the demyelination process.

It is interesting to identify factors that are responsible for structural destruction of myelin surrounding axons. Virus-mediated autoimmunity seen in MS and Theiler's virus infection was reported to cause T cell-mediated autoimmune disease related to demyelination [31,32]. Guillain-Barré syndrome, characterized by widespread dysfunction of peripheral nerves, may appear and cause acute inflammatory demyelinating polyneuropathy in JE patients [33]. According to our results, a myelin-specific autoimmune response might be a relatively important cause of demyelination among JE patients and with other viral infections [34,35].

The present study revealed that the proliferation of MBP-specific T-lymphocytes increases during JE viral infection. This likely induces a cascade of destruction of the axon-surrounding myelin. Because MBP-specific antibodies are also present in some asymptomatic mice, it seems that MBP is probably not the only target which can trigger autoimmunity against myelin. Other components of myelin including proteolipid protein (PLP) and myelin oligodendrocyte glycoprotein (MOG) are also known to be capable of eliciting specific antibodies in MS patients [36], suggesting the possibility of causing myelin destruction and the resulting demyelination. It was concluded that the JE virus which normally causes inflammation and neuronal degeneration in the CNS induces proliferation of specific T cells which mediate autoimmunity to destroy components of axon-surrounding myelin such as MBP.

## Competing interests

The authors declare that they have no competing interests.

## Authors' contributions

YFT performed all serological and immunological tests. CCW carried out electron microscopy. CKC was responsible for virus propagation. SKL guided all immunological works. WJC designed the whole study and wrote the manuscript. All authors were involved in reviewing and updating the text associated with the manuscript. All authors have read and approved the final manuscript.
